# Prospective Study of Optimal Obesity Index Cut-Off Values for Predicting Incidence of Hypertension in 18–65-Year-Old Chinese Adults

**DOI:** 10.1371/journal.pone.0148140

**Published:** 2016-03-02

**Authors:** Qian Ren, Chang Su, Huijun Wang, Zhihong Wang, Wenwen Du, Bing Zhang

**Affiliations:** National Institute for Nutrition and Health, Chinese Center for Disease Control and Prevention, Beijing, China; University of Bologna, ITALY

## Abstract

**Background:**

Overweight and obesity increase the risk of elevated blood pressure; most of the studies that serve as a background for the debates on the optimal obesity index cut-off values used cross-sectional samples. The aim of this study was to determine the cut-off values of anthropometric markers for detecting hypertension in Chinese adults with data from prospective cohort.

**Methods:**

This study determines the best cut-off values for the obesity indices that represent elevated incidence of hypertension in 18–65-year-old Chinese adults using data from the China Health and Nutrition Survey (CHNS) 2006–2011 prospective cohort. Individual body mass index (BMI), waist circumference (WC), waist:hip ratio (WHR) and waist:stature ratio (WSR) were assessed. ROC curves for these obesity indices were plotted to estimate and compare the usefulness of these obesity indices and the corresponding values for the maximum of the Youden indices were considered the optimal cut-off values.

**Results:**

Five-year cumulative incidences of hypertension were 21.5% (95% CI: 19.4–23.6) in men and 16.5% (95% CI: 14.7–18.2) in women, and there was a significant trend of increased incidence of hypertension with an increase in BMI, WC, WHR or WSR (*P* for trend < 0.001) in both men and women. The Youden index indicated that the optimal BMI, WC, WHR, WSR cut-off values were 23.53 kg/m^2^, 83.7 cm, 0.90, and 0.51 among men. The optimal BMI, WC, WHR, WSR cut-off values were 24.25 kg/m^2^, 79.9 cm, 0.85 and 0.52 among women.

**Conclusions:**

Our study supported the hypothesis that the cut-off values for BMI and WC that were recently developed by the Working Group on Obesity in China (WGOC), the cut-off values for WHR that were developed by the World Health Organization (WHO), and a global WSR cut-off value of 0.50 may be the appropriate upper limits for Chinese adults.

## Introduction

The prevalence of hypertension in adults aged 25 years or older was around 40% in 2008 worldwide[1]. The global prevalence of hypertension has been dramatically increasing for the past 2 decades[2]. Raised blood pressure is estimated to cause 7.5 million deaths and about 12.8% of the total mortality globally[1]. Hypertension meanwhile accounts for 57 million disability adjusted life years (DALYS) and accounts for 3.7% of total DALYS[1]. The World Health Organization (WHO) has estimated that hypertension directly causes about 62% of cardiovascular diseases (CVDs) and 49% of ischemic heart disease (IHD) globally[1].

It is generally recognized that overweight and obesity are major independent risk factors for hypertension[3–5], and the prevalence of overweight and obesity is increasing worldwide[6]. With modernization over the past three decades, overweight and obesity have increased tremendously in China[7].

Different anthropometric measures for obesity such as body mass index (BMI), waist circumference (WC), waist:hip ratio (WHR) and waist:stature ratio (WSR) have been proposed to define obesity[4].

Some studies have identified links between BMI and the risk of hypertension[8–10], and some other studies have also reported associations between abdominal accumulation of body fat with incidence of hypertension[11–13]. However, there is controversy regarding which anthropometric measure best defines obesity and conveys the highest risk for hypertension[4]. Most of the previous investigations of the association between these anthropometric measures for obesity and hypertension have been based on cross-sectional comparisons[4, 14–18].

Widely used among Western populations, BMI cut-off values of 25 and 30 kg/m^2^ for overweight and obesity, respectively, have been recommended by the WHO as international criteria for body fatness at the population level[8]. But it is clear that, it is not appropriate to use a single cut-off value to define obesity for all populations, for there are potential ethnic variation in body build and composition, and variation in the health risk associated with obesity among populations[19]. Some researchers suggested that country-specific and ethnic-specific BMI cut-off values for Asians are needed[20], for Asians have a higher body fat percentage than whites at the same BMI level[21]. Many studies in Asia have showed that Asian populations may require a lower cut-off values for BMI and WC compared with western populations to define obesity and abdominal obesity respectively[19].

In our prospective cohort study, we examined the association between levels of obesity indices (BMI, WC, WHR and WSR) and incidence of hypertension and used a receiver operating characteristic (ROC) curve analysis to assess the optimal cut-off values of these anthropometric indicators for overweight or obesity in Chinese adults. To our best knowledge, this is the first prospective study to use ROC curve analyses to identify optimal BMI, WC, WHR and WSR cut-off values for incidence of hypertension in Chinese adults sample.

## Subjects and Methods

### Study sample

The study uses two waves of data collected in 2006 and 2011 by the China Health and Nutrition Survey (CHNS) with representative samples drawn from nine provinces in China (Heilongjiang, Liaoning, Shandong, Jiangsu, Henan, Hubei, Hunan, Guizhou and Guangxi).

The CHNS is an ongoing study established in 1989, and it examined the nutritional status and health indicators of the Chinese population affected by the socioeconomic transformation of Chinese communities and society. Detailed descriptions of the study design and other information have been presented elsewhere[22]. Data sets and questionnaires can be downloaded from the CHNS Web sites (http://www.cpc.unc.edu/china). This research has been approved by the Institutional Review committees of the University of North Carolina at Chapel Hill and the National Institute for Nutrition and Health, Chinese Center for Disease Control and Prevention. All participants gave written informed consent for their participation in the survey.

Of the 5402 participants aged 18 to 65 years in 2006 who were involved in both surveys, and who were men, non-pregnant or non-lactating women in 2006, 4027 (75%) had complete and plausible measurements of blood pressure and anthropometric measurements, like weight, height, WC and hip circumference (HC). For example, 5-y changes in BMI < 10 kg/m^2^, and in height < 10 cm; a baseline BMI of 15–40 kg/m^2^, WC of 45–150 cm, HC of 55–155 cm, and WHR of 0.6–1.3; the difference between any two of the three measurements of systolic blood pressures (SBP) in each survey < 10 mm Hg, and the difference between any two of the three measurements of diastolic blood pressures (DBP) in each survey < 10 mm Hg[8]. Of the 4027 participants, 3253 (81%) with normal blood pressure in 2006 were included in our longitudinal sample.

We only included adults who were 18 to 65 years old, including nonpregnant and nonlactating women for teenagers, older persons, or pregnant or lactating women require different obesity indices cut-off values[8]. The exclusion of participants with extreme or implausible values in anthropometric measures or blood pressure helped us to increase the estimate precision.

### Measures

All participants underwent a standardized examination including the collection of SBP and DBP. Three blood pressure measurements were taken on the right arm by trained health workers, all of the workers followed the standardized procedure using regularly calibrated mercury sphygmomanometers with appropriate-sized cuffs. SBP was measured at the first appearance of a pulse sound (Korotkoff phase 1) and DBP was measured at the disappearance of the pulse sound (Korotkoff phase 5). Three measurements of SBP or DBP were averaged to reduce the effect of measurement error. Hypertension was defined as SBP ≥ 140 mm Hg, DBP ≥ 90 mm Hg or being diagnosed by a doctor previously [14].

Cumulative incidence was calculated by dividing new hypertension cases over the study period by the total at risk population in 2006.

Anthropometric measures were taken after the participants had removed their shoes and heavy clothing. Height, weight, WC and HC were measured by the trained workers. BMI (kg/m^2^) was calculated based on weight and height that were measured by trained workers who followed standardized procedures; while the participants breathed out gently, WC (cm) was measured at a point midway between the lower rib margin and the iliac crest; HC was taken at the level of maximal gluteal protrusion. WHR and WSR were calculated as WC(cm)/HC(cm) and WC(cm)/stature(cm) respectively[23].

Covariates such as age, sex, place of residence, smoking habits and alcohol consumption were collected by direct interviews, we calculated daily salt intake based on diet data using weighing methods in combination with 3 consecutive 24-h dietary recalls.

### Statistical analysis

We used the 2-tail independent *t* test or chi-square test to test differences between males and females in the means or proportions of baseline variables such as age, SBP, DBP, BMI, WC, WHR, WSR, smoking habits, alcohol consumption, place of residence and daily salt intake.

BMI, WC, WHR, and WSR were divided into quartiles. To determine whether these obesity indices were associated with incidence of hypertension, the Poisson regression model was used and modeled for participants after adjustment for age, smoking habits, alcohol consumption, place of residence and daily salt intake at baseline[8, 14]. *P* for trend was also calculated. To make them comparable with other studies, we stratified our analyses by sex.

To identify the obesity index that best predicted incidence of hypertension, we plotted ROC curves for each obesity index. The ROC curve is an analytical approach that has been widely used to determine cut-off values for decision making[8, 14, 21]. We compared the areas under the ROC curve (AUC) among men and women. AUC shows the ability of a test to correctly classify the participants with and without the disease, and the AUC values are usually used to compare overall performances of different screening tests[14]. In our study, AUC values were estimated by logistic regression models. To make our results comparable with other studies, we stratified our analyses by sex in crude and age-adjusted models.

To determine the optimal cut-off values for the obesity indices, the Youden index was calculated (sensitivity + specificity−1), and the values for the maximum of the Youden index was considered as the optimal cut-off points.

All statistical analyses were conducted using SAS 9.2 (SAS Institute, Cary, North Carolina, USA).

## Results

At baseline, the mean SBP and DBP were higher among men (116.7 mmHg and 76.6 mmHg, respectively) compared with women (113.0 mmHg and 74.0 mmHg; *P* < 0.001). Women had a higher mean BMI (23.0 kg/m^2^) compared with men (22.8 kg/m^2^) but the distribution of BMI was same among men and women. Men had higher means of WC and WHR, but smaller mean of WSR compared with women, and men and women had similar mean of HC. The proportions of Chinese men who were smokers (65.5%) and alcohol drinkers (61.9%) were much higher than those of women (2.7% and 8.9%, respectively). The distribution of daily salt intake was different among men and women (Table 1).

**Table 1:** Characteristics of 18- to 65-y-old, normotensive participants in 2006^1^.

	Men (*n* 1441)		Women (*n* 1812)		
	Mean or %	95% CI	Mean or %	95% CI	*P* value^2^
Age (years)	45.4	44.9, 46.0	45.6	45.1, 46.0	0.700
SBP (mm Hg)	116.7	116.2, 17.3	113.0	112.5, 113.5	<0.001
DBP (mm Hg)	76.6	76.3, 77.0	74.0	73.7, 74.4	<0.001
BMI (kg/m^2^)	22.8	22.6, 22.9	23.0	22.9, 23.2	0.027
<18.5 kg/m^2^ (%)	5.0	3.9, 6.1	5.6	4.5, 6.6	0.190
18.5- kg/m^2^ (%)	50.4	47.8, 53.0	47.2	44.9, 49.5	
23- kg/m^2^ (%)	22.7	20.5, 24.9	22.6	20.7, 24.5	
25- kg/m^2^ (%)	21.9	19.8, 24.1	24.7	22.7, 26.6	
WC (cm)	81.5	81.1, 82.0	78.7	78.3, 79.1	<0.001
HC (cm)	92.7	92.4, 93.1	92.8	92.5, 93.1	0.780
WHR	0.88	0.88, 0.88	0.85	0.84, 0.85	<0.001
WSR	0.49	0.49, 0.49	0.50	0.50, 0.51	<0.001
Ever smoked cigarettes (%)	65.5	63.1, 68.0	2.7	1.9, 3.4	<0.001
Alcohol drinker (%)	61.9	59.4, 64.4	8.9	7.6, 10.2	<0.001
Urban residence (%)	29.6	27.2, 31.9	28.5	26.5, 30.6	0.520
Daily salt intake (g/d)					
<6 g/d (%)	21.2	19.0, 23.4	30.4	28.2, 32.6	<0.001
6- g/d (%)	51.8	49.2, 54.5	49.1	46.7, 51.5	
12- g/d (%)	17.9	15.8, 19.9	12.7	11.1, 14.3	
18- g/d (%)	9.2	7.6, 10.7	7.9	6.6, 9.2	

SBP, systolic blood pressure; DBP, diastolic blood pressure; BMI, body mass index; WC, waist circumference; HC, hip circumference; WHR, waist:hip ratio; WSR, waist:stature ratio.

^1^ Values are means or percentages with 95% CI, *n* = 3253 (excluded participants with implausible anthropometric indices, e.g. WC <45 or >150 cm).

^2^ Men compared with women (independent *t* test for continuous variables or *x*^*2*^ test for categorical variables).

**Fig 1:**
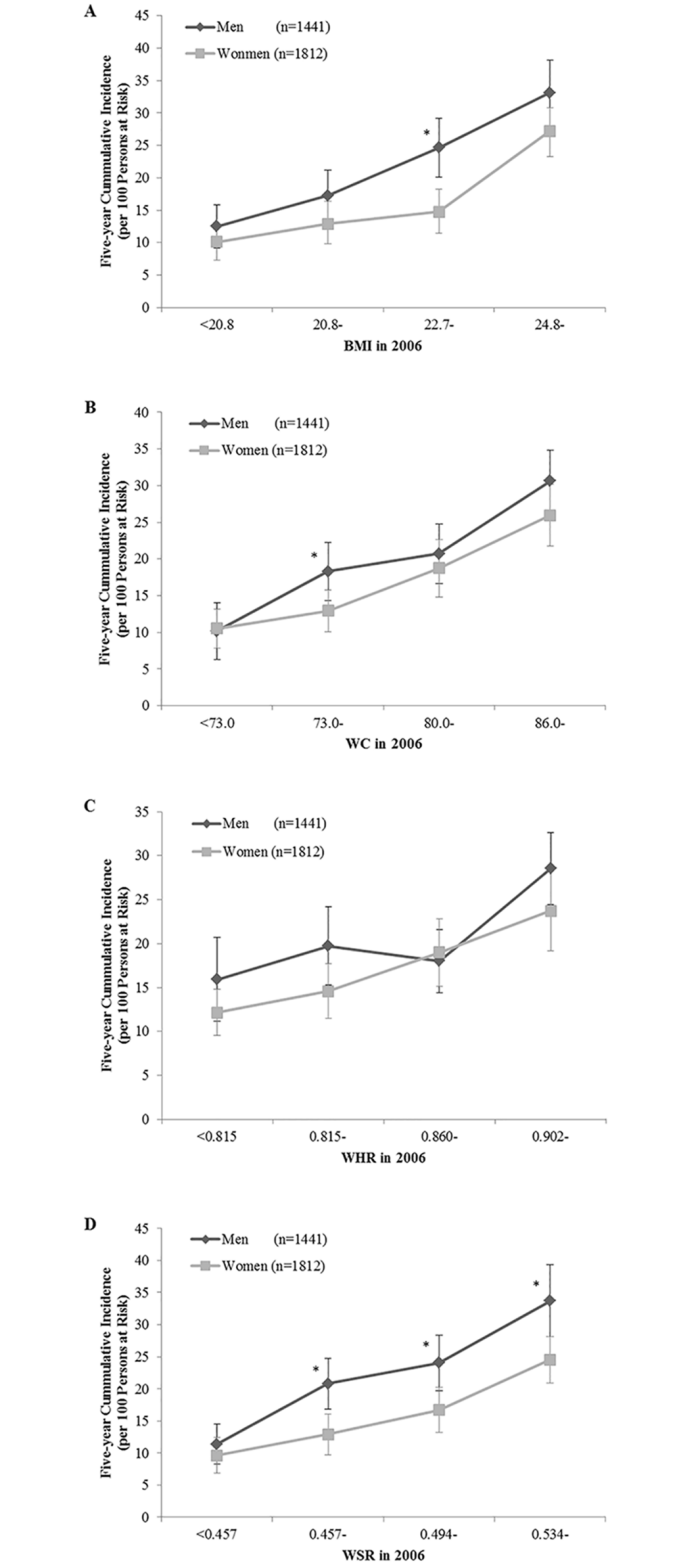
Five-year cumulative incidence and 95% CI of hypertension in Chinese adults (new cases per 100 persons at risk) by levels of (A) BMI, (B) WC, (C) WHR, (D) WSR in 2006. * Different from women, *P* < 0.05 chi-square test; *P*-trend < 0.001 for all.

5-y cumulative incidences of hypertension in men were 21.5% (95% CI: 19.4–23.6) and 16.5% (95% CI: 14.7–18.2) in women. There was a significant trend of increased incidence of hypertension with an increase in BMI, WC, WHR or WSR (*P* for trend < 0.001) in both men and women (Fig 1).

Table 2 shows the association between levels of obesity indices and incidence of hypertension. There was a significant trend of increased incidence of hypertension with an increase in BMI, WC, WHR or WSR (*P* for trend < 0.001) in both men and women. During the 5-year follow-up, the numbers of new cases of hypertension among people in the highest quartiles of BMI, WC, WHR, and WSR at the baseline examination were different from those in the lowest quartiles (*P* < 0.05).

**Table 2:** Obesity indices and incidence of hypertension.

	Men						Women					
	Range		Cases	No. of subjects	*P* value^1^	*P* trend^2^	Range		Cases	No. of subjects	*P* value	*P* trend
BMI (kg/m^2^)						<0.001						<0.001
Q1	<	20.7	42	357	-		<	20.8	44	436	-	
Q2	20.7	-	65	370	0.021		20.8	-	59	458	0.092	
Q3	22.6	-	81	349	<0.001		22.7	-	70	471	0.038	
Q4	24.6	-	122	365	<0.001		25.0	-	125	447	<0.001	
WC (cm)						<0.001						<0.001
Q1	<	75.0	41	329	-		<	72.0	41	416	-	
Q2	75.0	-	71	368	0.018		72.0	-	61	461	0.343	
Q3	81.0	-	82	383	0.012		78.0	-	79	482	0.054	
Q4	87.1	-	116	361	<0.001		84.6	-	117	453	<0.001	
WHR						<0.001						<0.001
Q1	<	0.835	58	359	-		<	0.802	45	451	-	
Q2	0.835	-	71	356	0.191		0.802	-	68	446	0.181	
Q3	0.879	-	81	369	0.057		0.844	-	83	478	0.108	
Q4	0.916	-	100	357	0.002		0.889	-	102	437	0.009	
WSR						<0.001						<0.001
Q1	<	0.453	39	366	-		<	0.460	43	454	-	
Q2	0.453	-	71	355	0.006		0.460	-	60	453	0.213	
Q3	0.487	-	87	357	<0.001		0.499	-	79	451	0.052	
Q4	0.524	-	113	363	<0.001		0.545	-	116	454	<0.001	

BMI, body mass index; WC, waist circumference; WHR, waist:hip ratio; WSR, waist:stature ratio.

^1^ Poisson regression models were used, adjusting for age, smoking habits, alcohol consumption, place of residence and daily salt intake.

^2^ Chi-square tests were used.

**Fig 2:**
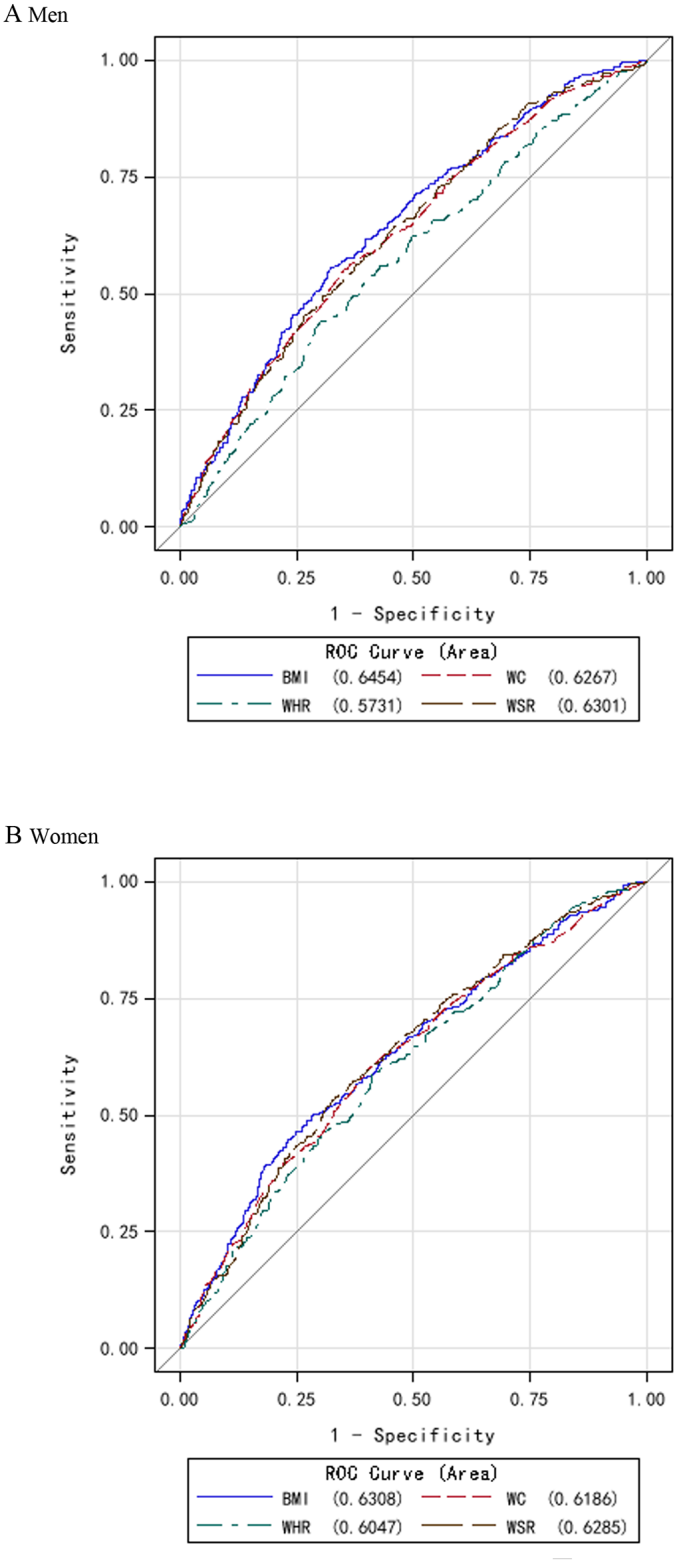
ROC curves for obesity indices in relation to incidence of hypertension. ROC, receiver operating characteristic; BMI, body mass index; WC, waist circumference; WHR, waist:hip ratio; WSR, waist:stature ratio.

To identify the obesity index that best predicted incidence of hypertension, the ROC curves and the AUC of the obesity indices in relation to incidence of hypertension were plotted and calculated (Fig 2). In predicting incidence of hypertension, the measures of BMI, WC and WSR tended to yield higher AUCs than did WHR. Among men, the AUC for BMI was 0.65 and the AUCs for WC, WHR, and WSR were 0.63, 0.57, and 0.63, respectively. Among women, the AUC for BMI was 0.63 and the AUCs for WC, WHR, and WSR were 0.62, 0.60, and 0.63, respectively.

Models with WHR had about 5–8% lower AUC compared with models with BMI for men (*P* < 0.001). Models with WC, WHR or WSR provided similar AUC compared with models with BMI for women (difference in AUC ≤ 3%; *P* > 0.05). BMI appeared to be a better discriminator than other obesity indices (WC, WHR and WSR) in predicting incidence of hypertension (wider AUC). When adding WC, WHR or WSR to a model with BMI, there was no increase in model fit (*P* > 0.05 in all of the models) (Table 3).

**Table 3:** Sex-specific AUC by different anthropometric indices for the prediction of hypertension.

		Men (*n* 1441)			Women (*n* 1812)		
Model	Independent variable	AUC^1^	95% CI	*P* value^2^	AUC	95% CI	*P* value
Crude model^3^	BMI	0.65	0.61, 0.68	-	0.63	0.59, 0.67	-
	WC	0.63	0.59, 0.66	0.184	0.62	0.58, 0.66	0.662
	WHR	0.57	0.54, 0.61	<0.001	0.60	0.57, 0.64	0.349
	WSR	0.63	0.60, 0.66	0.285	0.63	0.59, 0.67	0.938
	BMI + WC	0.65	0.61, 0.68	0.669	0.64	0.60, 0.67	0.874
	BMI + WHR	0.65	0.61, 0.68	0.879	0.64	0.61, 0.68	0.637
	BMI + WSR	0.65	0.61, 0.68	0.490	0.64	0.60, 0.68	0.723
Age-adjusted^4^	BMI	0.68	0.65, 0.71	-	0.72	0.69, 0.76	-
	WC	0.67	0.64, 0.70	0.250	0.71	0.67, 0.74	0.528
	WHR	0.63	0.59, 0.66	<0.001	0.70	0.66, 0.74	0.357
	WSR	0.66	0.63, 0.70	0.097	0.71	0.67, 0.74	0.506
	BMI + WC	0.68	0.65, 0.71	0.413	0.72	0.69, 0.76	0.997
	BMI + WHR	0.68	0.65, 0.71	0.573	0.72	0.69, 0.76	0.996
	BMI + WSR	0.68	0.65, 0.71	0.284	0.72	0.70, 0.75	0.987

AUC, area under the receiver operating characteristic curve; BMI, body mass index; WC, waist circumference; WHR, waist:hip ratio; WSR, waist:stature ratio.

^1^ AUC values were estimated by logistic regression models; range from 0.5 (no prediction) to 1.0 (perfect prediction).

^2^ Models compared with a model with BMI.

^3^ Crude models: include independent variables in the list.

^4^ Age-adjusted models: include independent variables and age.

Table 4 shows the optimal cut-off values for predicting incidence of hypertension. The Youden index indicated that the optimal BMI cut-off value was 23.53 kg/m^2^ (sensitivity, 0.555; specificity, 0.676), the optimal WC cut-off value was 83.7 cm (sensitivity, 0.552; specificity, 0.648), the optimal WHR cut-off value was 0.90 (sensitivity, 0.442; specificity, 0.699), and the optimal WSR cut-off value was 0.51 (sensitivity, 0.497; specificity, 0.693) among men. The optimal BMI cut-off value was 24.25 kg/m^2^ (sensitivity, 0.503; specificity, 0.715), the optimal WC cut-off value was 79.9 cm (sensitivity, 0.601; specificity, 0.595), the optimal WHR cut-off value was 0.85 (sensitivity, 0.591; specificity, 0.585), and the optimal WSR cut-off value was 0.52 (sensitivity, 0.534; specificity, 0.676) among women. The Youden index for BMI was highest in men and women among the measured obesity indices.

**Table 4:** Optimal obesity index cutoffs for predicting incidence of hypertension.

	Cutoff	Sensitivity	Specificity	Youden index
Men				
BMI	23.53 kg/m^2^	0.555	0.676	0.231
WC	83.70cm	0.552	0.648	0.200
WHR	0.90	0.442	0.699	0.141
WSR	0.51	0.497	0.693	0.190
Women				
BMI	24.25 kg/m^2^	0.503	0.715	0.218
WC	79.90 cm	0.601	0.595	0.196
WHR	0.85	0.591	0.585	0.176
WSR	0.52	0.534	0.676	0.209

BMI, body mass index; WC, waist circumference; WHR, waist:hip ratio; WSR, waist:stature ratio.

## Discussion

To our best knowledge, this is the first prospective study to use ROC curve analyses to identify optimal BMI, WC, WHR and WSR cut-off values for incidence of hypertension in Chinese adults sample.

A number of studies have shown that, as compared with Caucasians, Asians have a higher percentage of body fat at lower BMI and WC[24]. Indeed, it has been demonstrated that Asians are predisposed to visceral or abdominal obesity, and that the increased risks in Asians associated with obesity occur at lower BMIs[25]. WHO recommended BMI cut-off values for defining overweight and obesity were 25.0–29.9 kg/m^2^ for overweight, and 30 kg/m^2^ or higher for obesity, respectively[19]. These cut-off values may not be appropriate in Asians[25]. Many studies in Asia have demonstrated that Asians may require lower cut-off values for BMI and WC to define obesity compared with western populations[19], and the current definitions of overweight, obesity and central obesity based on Western populations need to be modified for the Chinese population.

In our prospective cohort study, we examined the association between levels of obesity indices and incidence of hypertension and used ROC analysis to assess the optimal cut-off values of these anthropometric indicators for overweight or obesity in Chinese adults.

Our findings showed that there was a significant trend of increased incidence of hypertension with an increase in BMI, WC, WHR or WSR (*P* for trend < 0.001) in both men and women. This finding was similar to results from Tuan’s study[14]. During the 5-year follow-up, the numbers of new cases of hypertension among people in the highest quartiles of BMI, WC, WHR, and WSR at the baseline examination were different from those in the lowest quartiles (*P* < 0.05).

The positive correlation between BMI and increased blood pressure could be explained by the increase in BMI is associated with the increase in body fluid volume, in peripheral resistance and in cardiac output. Increased blood pressure related to increased WC, WHR or WSR could be explained by the increase in visceral fat that is associated with the increase in leptin and insulin resistance and worse lipid profiles[14].

In our study, the optimal cut-off values for men and women were approximately 23.53 and 24.25 kg/m^2^ for BMI, 83.7 and 79.9 cm for WC, 0.90 and 0.85 for WHR, and 0.51 and 0.52 for WSR, respectively. In this population-based cohort of 18- to 65-y-old Chinese adults, our findings shows that the presence of overall obesity and central obesity increased the incidence of hypertension and that BMI appeared to be a better discriminator than other obesity indices (WC, WHR and WSR) in predicting incidence of hypertension (wider AUC). It also supports the idea that the combination of BMI and other obesity indices (WC, WHR and WSR) identify no better discriminator of incidence of hypertension than BMI alone in both sexes (*P* > 0.05).

The Working Group on Obesity in China (WGOC) has recently developed the cut-off level for overweight (24.0 kg/m^2^) using BMI for the general Chinese population[26–28]. In the present study, the optimal cut-off values for Chinese men and women adults were found to be 23.53 and 24.25 kg/m^2^ for BMI. These values were very similar compared with WGOC definitions in both men and women. The WGOC has also developed the cut-off values for overweight using WC for the general Chinese population, and the cut-off points are 85.0 cm for men and 80.0 cm for women[26, 27]. In our study, the optimal cut-off values for Chinese males and females adults were found to be 83.7 cm and 79.9 cm for WC, and these values were also very similar with WGOC definitions in both sexes. According to WHO criteria, WHR > 0.9 in males and > 0.85 in females denote abdominal obesity[29]. In our study, the optimal cut-off values for WHR for Chinese men and women adults were found to be 0.9 and 0.85. Our study has suggested that these WHR cut-off values could be applied to Chinese adults. Shao’s results indicated that WSR might be an optimal anthropometric indicator of metabolic syndrome risk factors, the cut-off value of WSR was approximately 0.50 in both sexes of Chinese adults[30]. In addition, several studies on CVD outcomes have suggested that a WSR cut-off value of 0.5 could be applied to both sexes, and there is a simple message: to keep your WC to less than half of your height to keep away from health risks[31–33]. The optimal cut-off values for men and women were approximately 0.51 and 0.52 for WSR in our study. Our study also supports this idea.

Some studies in other Asian countries have reported different results. In Gupta’s study, the BMI cut-off values to predict hypertension were 22.8 kg/m^2^ in males and 28.8 kg/m^2^ in famales, the optimal WC cut-off values varied from 91–92 cm in both males and females, the WHR cut-off values were about 0.90 in males and 0.78 in females respectively, and the optimal WSR cut-off values were 0.56 in males and 0.43 in females[15]. In Midha’s study, the cut-off points of BMI for predicting incidence of hypertension were identified as ≥ 24.5 kg/m^2^ in males and ≥ 24.9 kg/m^2^ in females, and the cut-off points for WC for predicting incidence of hypertension were estimated as ≥ 83 cm for males and ≥ 78 cm for females[1]. In Shabnam’s study, the cut-off points of WC according to maximum sum of sensitivity and specificity for detecting hypertension in men were 89.7 cm and in women were 93.9 cm, the cut-off points of BMI according to maximum sum of sensitivity and specificity for detecting hypertension in men were 25.7 kg/m^2^ and in women were 26.9 kg/m^2^[34]. To explain the difference in these obesity indices cut-off values, ethnicity may play an important role in determining the predictive power for hypertension. Different ethnicities often differ in socioeconomic status, cultural factors, lifestyles, food habits and physical activity levels, and ethnic groups may have different combinations of genes that are associated with hypertension and gene-environment interactions that may lead to the variation in blood pressure[8]. Other possible explanations are that these results were all based on cross-sectional studies and some of the studies were not conducted on a huge sample.

Dong et al. reported that BMI showed a better association than WC and WSR with hypertension in Chinese men[35]. In another study, Zhou et al. reported that hypertension was associated with different obesity indices in men and in women and the best indicator for hypertension was BMI in Chinese women[36]. In Gupta’s study, their results indicated that BMI was the best predictor of having hypertension[15]. These are agreement with the results of the analyses carried out in the present study. Some studies showed that WC was the best predictor of hypertension[37, 38], and some other studies showed that WSR was the best predictor of incidence of hypertension[39, 40]. To explain the difference in these results, ethnicity may play an important role. Further longitudinal studies are needed to determine these findings.

In addition, our study has suggested that the combination of BMI and WC, BMI and WHR or BMI and WSR has no better predictive power for hypertension than BMI alone (*P* > 0.05). However, to our best knowledge, this is the first prospective study to use ROC curve analyses to identify optimal BMI, WC, WHR and WSR cut-off values for incidence of hypertension in Chinese adults sample, further longitudinal studies are needed to determine these results, and further tries of proposing synthetically scores using the combined information of the four obesity measurements to quantify body shape (chilli, pear, apple and pear-apple) are needed. The possible explanation for the finding that WC, WHR and WSR did not perform better or add to the prediction of incidence of hypertension by BMI in Chinese is that compared with other races and ethnicities, this population accumulate more total body fat and visceral fat with the increase of body weight[14].

Compared with obesity indicators with WC, BMI appears to be sufficient because that BMI is collected more often in health and nutrition surveys, interventions and clinics, and collected with universally accepted protocols and easier to interpret[14].

This study has several limitations. First, although obesity indices might change due to lifestyle modification during the 5-year follow-up period, only measurements in 2006 were used in this analysis, and changes in individual obesity were not considered, further studies should assess the association between the changes in individual obesity indices and incidence of hypertension. Second, AUC is widely used as the measure of a diagnostic test’s discriminatory power and the maximum value for the AUC is 1.0[41]. An AUC value of 1.0 indicates a (theoretically) perfect test (i.e., 100% sensitive and 100% specific), and an AUC value of 0.5 indicates no discriminative value (i.e., 50% sensitive and 50% specific)[41]. There are several scales for the interpretation of AUC but, generally, ROC curves with an AUC of 0.97 has a very high clinical value and an AUC ≤ 0.75 are not clinically useful[41]. The AUC values of our study range from 0.57 to 0.72, which are not high enough for clinically use, further tries of proposing synthetically scores using the combined information of the four obesity measurements to quantify body shape are needed to obtain a larger AUC value. Further studies should assess the association between overall/central obesity and hypertension using different methods. The moderate levels of AUC suggest that other factors may also contribute to the prediction of incidence of hypertension, the BMI, WC, WHR and WSR cut-off values based on the sensitivity-specificity approach are considered as useful thresholds to define overweight for public health but are not considered as screening levels for incidence of hypertension[8].

The present study investigated the associations between obesity indices and incidence of hypertension in a large population of Chinese adults sample. The present study used a large sample to estimate optimal cut-off values to predict incidence of hypertension. These data are appropriate for defining cut-off values of BMI, WC, WHR and WSR in Chinese adults.

In conclusion, the present study showed that BMI may be better than other indicators of obesity for discriminating incidence of hypertension. Our data suggest that, the cut-off values for BMI and WC that were recently developed by WGOC, the cut-off values for WHR that were developed by WHO, and a global WSR cut-off value of 0.50 may be appropriate upper limits for Chinese adults.
